# The Relationship Between Game-Related Assessment and Traditional Measures of Cognitive Ability—A Meta-Analysis

**DOI:** 10.3390/jintelligence12120129

**Published:** 2024-12-16

**Authors:** Tanja Bipp, Serena Wee, Marvin Walczok, Laura Hansal

**Affiliations:** 1 Department of Psychology, Heidelberg University, 69117 Heidelberg, Germany; marvin.walczok@psychologie.uni-heidelberg.de (M.W.); laurahansal@t-online.de (L.H.); 2 School of Psychological Science, The University of Western Australia, Crawley, WA 6009, Australia; serena.wee@uwa.edu.au

**Keywords:** cognitive ability, game-related assessment, gamification, construct validity, individual performance

## Abstract

Technological advances have introduced new methods for assessing psychological constructs, moving beyond traditional paper-pencil tests. Game-related assessments (GRAs) offer several advantages for research and practice, though questions about their construct validity persist. This meta-analysis investigated the relationship between indicators derived from computer-based games and traditional cognitive ability measures, examining whether measurement scope (single vs. multiple indicators) or measurement medium of cognitive ability (computer-based vs. paper-pencil) influences this relationship. We identified 52 eligible samples stemming from 44 papers, including data from over 6100 adult participants. The results from three-stage mixed-effects meta-analyses showed an overall observed correlation of *r* = 0.30 (*p* < 0.001; corrected *r* = 0.45) between GRA indicators and traditional cognitive ability measures with substantial heterogeneity in effect sizes. Stronger relationships were found when cognitive ability was measured by multiple indicators, but no differences emerged based on the measurement medium of cognitive ability. Furthermore, GRAs intended to assess cognitive ability did not show stronger relationships with traditional measures of cognitive ability than GRAs not specifically used to measure cognitive ability. Overall, our findings suggest that GRAs are related to traditional cognitive ability measures. However, the overall effect size raises questions about whether GRAs and traditional measures capture the same aspects of cognitive ability or if GRAs also measure other constructs beyond cognitive ability.

## 1. Introduction

Technological advancements revolutionize the possibility of investigating and measuring psychological constructs, such as cognitive ability, using assessments beyond traditional measures (e.g., paper-pencil tests). These technological advances include the use of game-related assessment (GRA), which refers to a wide range of games used for assessment purposes, including the integration of gaming mechanics in traditional measures, the specific development of games to measure the psychological construct of interest, or the use of commercially available games for assessment purposes (Landers and Sanchez 2022; Ramos-Villagrasa et al. 2022). Several authors have noted the potential advantages of using GRAs over traditional measures in applied settings. These benefits include reduced anxiety, less careless responding, lower chances of faking, and more positive applicant reactions (Weinder and Short 2018). However, other factors, such as practice effects or increased working memory demands during gameplay, could introduce systematic measurement errors, which may affect the reliability and validity of GRAs (Hawkes et al. 2018).

Support for the validity of GRAs is particularly scarce (Ramos-Villagrasa et al. 2022), and it remains unclear whether these assessments accurately measure constructs they intend to measure or if they inadvertently capture other knowledge, skills, abilities, and characteristics (KSAO) of applicants. For example, a GRA developed to assess facets of conscientiousness turned out to be a better predictor of cognitive ability than conscientiousness (e.g., Wu et al. 2022). Although an unintentionally measured KSAO may be job-relevant, its assessment by the GRA undermines the specificity and accuracy of the evaluation, thus posing a serious threat to the construct validity of GRAs. As previously highlighted, it is essential to distinguish between the methods used to assess a construct and the construct itself (Arthur and Villado 2008; Campbell and Fiske 1959; Tippins et al. 2021). For example, comparing paper-pencil and computer-based administration of cognitive ability measures, power-based tests appeared to be equivalent (*r* = 0.91), but speed tests were not (*r* = 0.72) (Mead and Drasgow 1993). Understanding this distinction helps ensure that assessments accurately capture the intended constructs without being confounded by the measurement method.

Despite concerns about construct validity, GRAs are increasingly used in organizational selection practices (Georgiou et al. 2019; Ohlms et al. 2024). This trend could potentially lead to incorrect personnel decisions, such as selecting candidates who may be poorly suited for the job. Thus far, most research on GRAs has focused on their validity in measuring personality (Ramos-Villagrasa et al. 2022), with limited empirical evidence of their validity for measuring cognitive ability. Interestingly, some studies indicate that scores from commercially available electronic games, though not designed for psychometric assessment, can validly predict cognitive ability (Quiroga et al. 2015). However, there is no systematic review and summary of the evidence for GRAs as cognitive ability assessments, representing a critical gap. This is particularly important given the extensive research showing the importance of cognitive abilities in predicting outcomes such as academic and job performance (Kuncel et al. 2004; Nye et al. 2022; Ones et al. 2012; Sackett et al. 2017, 2022).

To address this issue, we provide meta-analytic evidence on the relationship between GRA and traditional measures of cognitive ability. Additionally, we investigate whether (i) the number of measures used (one vs. multiple measures for cognitive ability, and similarly, one or more games for GRA) or (ii) the measurement medium for cognitive ability (paper-pencil vs. computer-based test) moderates the strength of the relationship between GRA and cognitive ability. By uncovering shared and non-shared variance of GRAs and traditional measures of cognitive ability in adult samples, our results indicate the extent to which modern approaches of GRA assess cognitive ability, laying the foundation for evidence-based practice in applied assessment contexts. These empirical contributions aim to pave the way for theoretical advancements in understanding the degree to which GRAs may intentionally and unintentionally measure cognitive ability, ultimately guiding their applications in real-world settings.

## 2. The Relationship Between GRA and Cognitive Ability

### 2.1. Cognitive Ability: From Traditional Measures to Game-Related Assessment

Cognitive ability is a multidimensional and hierarchically organized construct that consists of a general factor, often referred to as general mental ability (GMA at Stratum III), along with several broad ability factors (at Stratum II) and many narrower ability factors (at Stratum I) (Schneider and McGrew 2012). GMA represents a broad cognitive capacity underlying performance across various cognitive tasks, reflecting an individual’s overall intellectual capacity. Broad ability factors represent domain- or task-specific abilities covering a relatively broad domain, such as visual processing, short-term memory, or fluid reasoning, and narrow ability factors represent such abilities but in a much more circumscribed domain, such as reading or writing speed (McGrew 2009). The assessment of cognitive ability and the associated development of measures has a long tradition in psychological research (Sackett et al. 2017). Traditional approaches to assessing cognitive abilities include standardized psychometric tests, in which tasks are presented to individuals or groups in one test or combined test batteries (Sternberg 2020). For example, language-free matrices tests or complex test batteries like the Wechsler Intelligence Scales (WAIS) are used in a wide variety of fields; see Flanagan and McDonough (2018) for an overview of contemporary batteries and assessments or Stanek and Ones (2018) for an overview how various measures are mapped to different cognitive ability constructs. Besides these traditional paper-pencil tests, more process-oriented assessments have also been developed based on cognitive approaches, for example, focusing on assessing working memory capacity with variations in the complex span task (Ellingsen and Engle 2020). Given this long history of assessing cognitive ability, it is unsurprising that standardized cognitive ability measures have excellent assessment and measurement properties and are cost- and time-effective for use in practice (Dilchert 2018).

Nowadays, technological developments enable new assessment approaches (Adler et al. 2018). For instance, test-takers can complete traditional measures on their personal device in locations of their choosing and without being proctored (Tippins et al. 2006). As well as a greater variety of platforms, technological advances have opened up access to a greater variety of data, which may even allow us to measure cognitive ability without using traditional psychometric tests at all (Kantrowitz and Gutierrez 2018; Koch et al. 2021). One such modern technology-based assessment approach refers to game-related assessment (GRA) that encompasses the application of gamification in the assessment context (Ramos-Villagrasa et al. 2022). Gamification is broadly defined as “the use of game design elements in a non-game context” (Deterding et al. 2011, p. 9) and has also led to applications in personnel selection contexts. To better delineate the types of assessments encompassed by GRAs, Ramos-Villagrasa et al. (2022), building on the taxonomy by Landers and Sanchez (2022), proposed a comprehensive classification of GRAs along a continuum of playfulness, ranging from traditional assessments at the low end to playful games at the high end. Within this range, they identify three intermediate types of GRA: gamified assessments, gamefully designed assessments, and game-based assessments, each representing an increasing level of playfulness.

Gamified assessment involves adding game mechanics to existing (i.e., traditional) assessments. Game mechanics shape a player’s experience by defining what actions they can take, how they can take them, and what happens based on their actions (Landers and Sanchez 2022). As outlined in a taxonomy proposed by Hervas et al. (2017), game mechanics can be categorized into six main categories: goals, status, randomness, appointment, scoring, and immersion. Goals drive player actions, providing purpose and direction for their efforts during the game. Goals can be implemented through levels, quests, challenges, or milestones. Status differentiates players, often by using rankings or leaderboards to compare performance. Randomness introduces unpredictability (real or apparent) into the game, using elements such as lotteries, hidden surprises, or dynamic changes to keep the gameplay fresh and engaging. Appointment mechanics relate to time-specific conditions for player participation, like time-limited goals or scheduled reward delivery. Scoring mechanics provide feedback on player progress through points, bonuses, or other forms of acknowledgment for effort and achievement. Lastly, immersion mechanics help to deeply engage players by encouraging them to take on specific roles, follow storytelling narratives, or explore the game environment. The inclusion of at least one game mechanic, for example, the use of storytelling, progress feedback for participants, the achievement of badges through the assessment, or the use of leaderboards to compare performance (e.g., Fetzer et al. 2017; Landers et al. 2017; Weinder and Short 2018), is what transforms an existing traditional assessment into a gamified assessment.

Gamefully designed assessments are distinguished from gamified assessments by incorporating game mechanics from the outset of their development. That is, they are designed to be inherently playful—through the deliberate inclusion of at least one of these game mechanics.

Game-based assessments represent an even more comprehensive integration of game mechanics, requiring the inclusion of multiple game mechanics into the development of new assessments, which users experience within a core gameplay loop (Landers and Sanchez 2022). A core gameplay loop is the sequence of repeated actions that players engage in throughout the game. It typically involves a cycle of activities—such as exploring, completing challenges, receiving feedback, and improving skills—that create a sense of progression and maintain engagement. In game-based assessments, this loop involves participants performing assessment-related tasks, receiving feedback, and adjusting their strategies, providing a dynamic and immersive experience. This deep integration of mechanics and the central gameplay loop distinguishes game-based assessments from simpler gamified or gamefully designed assessments. Game-based assessments can be developed using either a theory-driven approach, which focuses on accurately measuring a theoretical construct (e.g., cognitive ability), or developed in a data-driven manner, which emphasizes the prediction of an intended criterion (e.g., job performance).

Finally, playful games refer to commercially available games originally designed for entertainment but later adapted for purposes such as assessment, education, or training. For example, Sanchez et al. (2022) examined whether a virtual reality game (i.e., Project M) could be used to assess emotional intelligence. Additionally, several authors investigated video games to enhance study participants’ cognitive ability (overview in Lumsden et al. 2016; Sala et al. 2018).^1^

GRAs have already been examined for their capacity to assess personality traits (e.g., Harman and Brown 2022), with several studies showing that a broad range of GRAs can, in addition, be related to cognitive ability. For instance, gamified assessments have been developed to cover various aspects of cognitive functioning by embedding different tasks into a storyline (e.g., placing the test-taker as the protagonist in an interactive story settled in space) (Chicchi Giglioli et al. 2018). There are also successful examples in the literature of theory-driven game-based assessments that have been specifically designed to measure cognitive ability. For instance, based on game design theory and the Cattell–Horn–Carroll (CHC) model (McGrew 2009), Landers et al. (2021) provided convincing empirical evidence that their game-based assessment, which included different minigames targeting specific cognitive abilities, correlated highly with a latent GMA factor (β = 0.97). Similarly, Ohlms et al. (2024) developed a Minecraft-based assessment where participants solve verbal, numerical, and figural problems embedded in a storyline. This game correlated *r* = 0.51 with a traditional paper-pencil test measuring these specific abilities. Interestingly, game-based assessments developed to measure other constructs (e.g., conscientiousness) were also shown to substantially predict cognitive ability (Wu et al. 2022). Furthermore, even playful games (e.g., Tetris, League of Legends), which were not developed to measure cognitive abilities, also correlate positively with scores on traditional cognitive ability measures (Weinder and Short 2018). Performance in such games, for example, in terms of overall game scores or different parameters while playing the game, correlates positively with cognitive test scores. For example, top scores obtained in Tetris correlated positively with a measure of spatial skills (e.g., Adams and Mayer 2012). Or, the number of completed levels or the total points achieved in commercial games on the iPad^®^ or Wii U^®^ correlated 0.79 on the latent level with a battery of cognitive ability tests targeting fluid reasoning, visuospatial ability, or processing speed (Quiroga et al. 2019). Trace data modeled from seven mini-games also predicted GMA scores (Auer et al. 2022).

Overall, there seems to be substantial overlap between traditional measures of cognitive ability and more recently developed GRAs. GRAs that have been explicitly designed to measure cognitive ability tend to align closely with traditional assessments of cognitive ability (e.g., Landers et al. 2021; Ohlms et al. 2024). But even GRAs not explicitly developed to measure cognitive ability show significant overlap with traditional cognitive ability measures, targeting GMA (e.g., Weinder and Short 2018; Wu et al. 2022) as well as specific cognitive abilities (e.g., Adams and Mayer 2012). However, the empirical evidence on the relationship is mixed, with varying effect sizes (e.g., Roman et al. 2024). Therefore, it is not yet clear how effective GRAs are, on average, at predicting scores in traditional assessments of cognitive ability. Furthermore, research and practice urgently need insights into the factors that moderate the relationship between GRAs and traditional measures of cognitive ability.

### 2.2. Expected Relationship and Moderating Factors Between GRA and Traditional Measures of Cognitive Ability

The relationship between GRAs and traditional measures of cognitive ability is generally expected to be positive because both are influenced by the same underlying construct of cognitive ability. Regardless of whether a game was developed to assess cognitive ability, both gaming and testing situations appear to tap into similar aspects of cognitive functioning. For example, players and test-takers are often required to solve abstract and novel problems (Roman et al. 2024), such that the scores obtained from games and tests should be positively related. Furthermore, tests and games often place similar demands on cognitive processes. Quiroga et al. (2015) concluded that brain games and puzzles tap into the same abilities measured by traditional ability tests. Also, van Lill et al. (2023) postulated that performance in games aiming at cognitive processes would be significantly influenced by cognitive abilities such as fluid reasoning or short-term memory. In fact, Baniqued et al. (2013) found that playing casual web-based games triggered the same reasoning and working memory abilities as did tests.

However, empirical studies have shown substantial variation in the strength of this relationship, with some studies also reporting nonsignificant relationships (Quiroga et al. 2015, 2011). Several reasons could explain the variation in this relationship. First, although playing a game and taking a cognitive ability test both tap into cognitive abilities, they might assess different abilities. Games often emphasize spatial or figural abilities, such as in fast-moving first-person shooting games, or place strong demands on working memory, such as in real-time strategy games where players manage resources, control military units, and adapt to changing game states. In contrast, traditional tests are more likely to focus on verbal or numerical abilities (e.g., measuring vocabulary, reading comprehension, and mathematical reasoning) (Hawkes et al. 2018; Weiner and Sanchez 2020). Additionally, video games may require specialized knowledge and tap into sensory and psychomotor abilities that are not often assessed by traditional cognitive ability tests (at least not to the same degree). These differences might lead to only moderate relationships between GRAs and traditional cognitive ability tests.

Second, the measurement approach itself might explain the varying relationships. GRAs offer a more dynamic, detailed, and engaging assessment method. They allow for the collection of real-time, behaviorally-oriented indicators as individuals interact with the game. This includes data such as reaction times, mouse movements, and action sequences within the game (Weinder and Short 2018). Such data may provide a richer, more nuanced understanding of cognitive processes and may be better aligned with process-oriented approaches to intelligence (Ackerman 2012). Additionally, the engaging nature of GRAs can help individuals stay focused and motivated, potentially leading to more accurate assessments of their cognitive ability. In contrast, traditional paper-and-pencil tests represent a more static approach to assessing cognitive ability. Tests, by using a fixed set of questions or tasks that do not change in response to the test-taker’s actions or answers, offer a limited snapshot of an individual’s abilities. Numerous traditional tests cannot adapt to the test-taker’s performance and may not fully capture their problem-solving processes or adaptability. They may miss important aspects of cognitive abilities that would be revealed through interactive and adaptive tasks.

Third, differences between the two approaches might be explained by constructs or effects that are irrelevant to cognitive ability but affect performance in GRA. For example, cognitive ability test scores differed when using different device types (i.e., desktop computer vs. mobile phone) even with the same measure of cognitive ability (Traylor et al. 2021). Therefore, concerning GRA, it can be expected that technical factors, such as device type, screen size, or connection rate while playing, substantially affect gaming performance (Hawkes et al. 2018). Further, gaming scores are affected by training or practice (Landers and Sanchez 2022; Quiroga et al. 2009). Other factors, such as player demographics, have also been suggested to affect gaming performance. Intuitively, gamers (vs. non-gamers) are expected to be better in maneuvering gaming environments (Foroughi et al. 2016), and there are concerns that women and older applicants might be disadvantaged by game-based assessments (Landers et al. 2021; Melchers and Basch 2022). Empirical evidence strongly suggests that performance in games is also linked to other constructs, such as personality traits (Hawkes et al. 2018; Weinder and Short 2018).

### 2.3. Aims and Hypothesis

The current meta-analysis investigates the strength of the relationship between GRAs and traditional measures of cognitive ability. In doing so, it aims to determine the effectiveness of using GRAs as an alternative assessment method for cognitive ability and to identify factors that may moderate this relationship.

First, considering the theoretical similarities and differences between GRAs and traditional measures of cognitive ability, we expected a positive relationship between the broad array of GRAs (encompassing serious and playful games) and traditional tests of cognitive ability.

**Hypothesis** **1.** *Indicators of individual performance from game-related assessment are positively related to traditional measures of cognitive ability.*

We also expected that this relationship is moderated by both the measurement scope (multiple vs. single measures) and the measurement medium used. For measurement scope, we expected a stronger relationship when either the GRA is based on multiple games or when cognitive ability is assessed by multiple (sub)tests compared to single games or tests. This expectation is based on the core principle that using repeated measures enhances the reliability of assessment and, therefore, validity (Hawkes et al. 2018). Multiple measures reduce the influence of measurement error, thereby providing more stable and accurate estimates. Further, multiple measures tend to provide a more comprehensive assessment. For example, multiple cognitive ability tests in a test battery can measure various specific abilities (e.g., fluid reasoning, comprehensive knowledge, visual processing). Similarly, GRAs from multiple games can capture a wider range of cognitive processes involved in different game scenarios (e.g., strategy games require fluid reasoning, puzzle games require visual processing, and trivia games require comprehension knowledge). By using multiple (vs. single) measures, both traditional tests of cognitive ability and GRAs can provide more reliable and comprehensive measurements, thereby strengthening the relationship between GRAs and cognitive ability. Our expectation is supported by prior findings for both assessment methods separately. Not only has using aggregated scores from multiple (sub)tests of different batteries been shown to enhance the reliability and validity of traditional cognitive ability assessments (Wahlstrom et al. 2012) but such an approach has also been successfully applied to GRAs by aggregating indicators from several games to a composite game score (e.g., Landers et al. 2021; Quiroga et al. 2015).

**Hypothesis** **2.** *The measurement scope moderates the positive relationships between game-related assessment and cognitive ability. (A) If multiple (sub)tests are used to assess cognitive ability, the relationship is stronger compared to when only one (sub)test is used. (B) If indicators from multiple GRAs are used, the relationship is stronger compared to when the indicator(s) from only one GRA are used.*

For the medium of assessment, we postulated that the approach to measuring cognitive ability influences the relationship. In addition to traditional paper-pencil tests, several test batteries have been adapted for computer administration. Various computer-based cognitive tasks have also been successfully used to assess specific abilities (e.g., working memory) (Ellingsen and Engle 2020). We expected a stronger relationship between computer-based GRAs and computer-based cognitive ability tests, as the same medium of assessment is used. The assumption is that using the same medium for both testing and playing imposes similar demands in both situations, leading to higher correlations. Mead and Drasgow (1993) have already provided insights into the role of the medium of assessment for cognitive ability. While they found no differences between computer-based and paper-pencil modes of administration for power tests, they did find differences for speeded tests of cognitive ability. They explained their finding by suggesting that other motor and coordination skills play a role in speed tests that are administered on the computer, when compared to paper-pencil tests, leading to a lower relationship. Given the higher degree of overlap between demands or required skills of a computer game and a computer-administered test, we expected that the relationship would be higher compared to a situation in which performance indicators stemming from a GRA are related to a paper-pencil cognitive ability test.

**Hypothesis** **3.** *The measurement medium moderates the relationships between GRA and cognitive ability in such a way that the relationship is stronger if the cognitive ability test is administered using a computer vs. by a paper-pencil test.*

In addition, we shed light on further factors that potentially influence the relationship between GRA and traditional measures of cognitive ability on an exploratory basis. First, we took a closer look at what kind of cognitive ability is assessed by the traditional measure. Given the ongoing discussion about the role of GMA compared to specific cognitive abilities to predict outcomes, for example, at work (e.g., Lang and Kell 2020), we tested whether the relationship between GRA and cognitive ability differs when cognitive ability is assessed at the highest level (GMA, Stratum III) vs. broad or narrow abilities (Stratum II/I) within the CHC model (Schneider and McGrew 2012).

- *Research Question 1*: Are there any differences in the strength of the relationship between GRA and traditional tests of cognitive ability based on whether general cognitive ability/GMA vs. broad and narrow abilities were measured?

Second, taking into account GRAs with varying degrees of playfulness (Ramos-Villagrasa et al. (2022), ranging from assessments intentionally designed to measure cognitive ability to commercially available games designed for entertainment, we examined two additional research questions about whether the purpose of the GRA influences the relationship. We tested whether the relationship differs when comparing existing games (e.g., commercially available video games) to GRAs specifically developed or adapted with another purpose than playing in mind (e.g., for the assessment of specific constructs or training purposes). We also examined whether GRAs specifically intended to measure cognitive ability correlate differently with traditional cognitive ability tests compared to GRAs with other purposes (assessing personality traits or GRAs used for training purposes). While it is reasonable to expect that GRAs theory-driven designed to measure cognitive ability would show relationships with traditional tests of cognitive ability (e.g., Landers et al. 2021), data-driven approaches might also lead to comparable correlations. This could happen if trace models are developed based on ability-based behavioral indicators extracted from the gaming environment (Auer et al. 2022).

- *Research Question 2*: Are there any differences in the strength of the relationship between GRA and traditional tests of cognitive ability based on (A) whether the GRA was based on existing game(s) vs. specifically developed or adapted game(s) and (B) whether the GRA was specifically designed to assess cognitive ability vs. serves other purposes?

## 3. Materials and Methods

### 3.1. Literature Search and Inclusion Criteria

**Figure 1:**
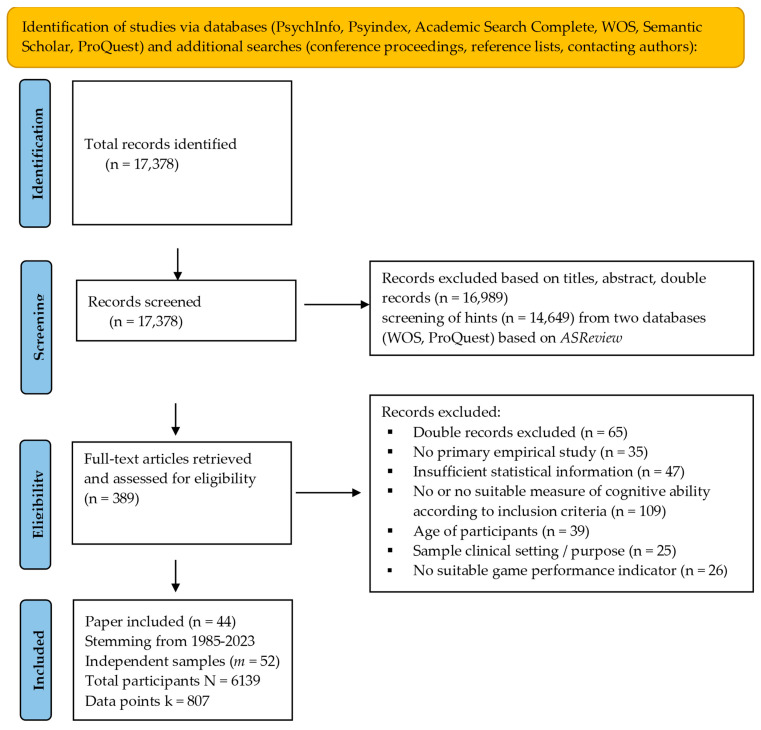
Illustration of the applied search process and screening criteria.

We used four different search strategies to identify relevant studies for inclusion in our meta-analysis. First, we searched five online databases “Academic Search Complete (1887-onwards)”, “APA PsycArticles (1984-onwards)”, “PsycInfo (1945-onwards)”, “Web of Science (Core collection, 1900-onwards)”, and “ProQuest (dissertation & thesis full text: The humanities and social sciences collection; 1985-onwards)”. In each database, we searched for titles and abstracts that satisfied two sets of criteria: one set for GRAs and one for cognitive ability. For the gaming part, we used the following search terms: “game-based”, “GBA”, “gamification”, “gamified”, “gameful”, “gamefully designed”, or “serious game”. For the cognitive ability part, we used the following search terms: “intelligence”, “general mental ability”, “cognitive ability”, “specific cognitive abilities”, “cognitive assessment”, “specific aptitudes”, “CHC theory”, “Cattell-Horn-Carroll theory”, “Gf-Gc theory”, “primary mental abilities”, “intelligence measures”, “g factor”, “IQ”, ”spatial ability”, or ”working memory”. Second, we screened conference programs of the Society of Industrial and Organizational Psychology (2004–2018) and the Academy of Management Meetings (1998–2023). Third, we examined reference lists from previous research in this area and searched specifically for studies from researchers who have been active in this area. We also used the AI-based tool “Semantic Scholar” to identify studies similar to key papers we had already identified for inclusion based on their high relevance and impact on the field. We limited these search strategies to identifying studies available up to the end of 2023. Lastly, we contacted authors to obtain all necessary information from relevant papers identified (e.g., containing potentially relevant data) and searched for additional information in the Open Science Framework. Specifically, we contacted n = 20 authors from the research field in which games are used to train cognitive abilities (cf. meta-analysis of Sala et al. 2018) and asked them to provide us with details (e.g., on the relationship between game scores and cognitive ability measures used at baseline/in the control groups). Based on our search strategy, we identified over 17,300 potentially relevant records (see PRISMA flowchart in Figure 1).

Next, we screened the identified records for inclusion in our meta-analysis based on the following criteria (summarized in Table 1). First, concerning study design and statistics, we included all empirical studies that reported correlations between indicators stemming from GRA and traditional measures of cognitive ability.

Second, we included the full range of GRAs as defined by Ramos-Villagrasa et al. (2022), ranging from gamified assessments to playful games used for assessment purposes. Specifically, we included all effect sizes where the GRA utilized at least one of the game mechanics described by Hervas et al. (2017). Consistent with previous reviews (Landers and Sanchez 2022; Ramos-Villagrasa et al. 2022), we focused on technology-based GRAs, excluding non-digital games such as board games or physical sports. Electronic games, such as Tetris or Big Brain Academy, were included (e.g., Adams and Mayer 2012). Additionally, the study needed to report an indicator of objective individual performance that was not influenced by the performance of others. For example, we included studies in which scores were directly obtained from people playing a game in the lab (Quiroga et al. 2016) or where participants self-reported their objective game scores in online games (e.g., self-report rank for Counterstrike) (e.g., Cretenoud et al. 2021). Further, we excluded studies that (a) only used a game frame (e.g., presenting a traditional cognitive measure as a game without implementing any game mechanics); (b) relied solely on subjective gaming indicators that do not directly capture game performance (e.g., prior gaming experience, or self-reports of playing frequency); and (c) involved non-computer-based, zero-sum games or multiplayer interactions (e.g., board games like Taboo played in pairs or team sports like soccer).

Third, concerning cognitive ability, the measures used in the primary studies had to be an instrument assessing a construct that we could locate in one of the three levels of the CHC model (McGrew 2009). For example, we included paper-pencil or computer-administered measures of cognitive ability or established measures of working memory or attentional control (e.g., measured by the Operation span task) (e.g., Atkins et al. 2014) but excluded measures to diagnose brain damage, cognitive deficits, or dementia (e.g., Montreal-Cognitive-Assessment-Test), or other measures stemming from biological approaches (e.g., indicators stemming from EEG research).

Fourth, to be included in our final dataset, a study needed to report the sample size and the nature of the sample. As we were interested in potential applications of our meta-analytic findings to the employment context, we focused on healthy adult samples and selected studies with participants 18 years or older. Thus, we excluded samples that included younger participants (e.g., kindergarten or school children) or studies with samples stemming from clinical settings (e.g., dementia or care patients).

For screening the records identified for eligibility for our inclusion criteria, we combined traditional screening methods and modern technological approaches. That is, for four of the six databases, the authors of this study manually screened the titles and abstracts of the identified records. For the remaining databases (WOS and ProQuest), we used the open-source tool AS Review to aid with the screening. AS Review is an AI-based tool that helps to conduct systematic reviews by supporting the screening process based on an active machine learning algorithm. The AS Review software is freely available at https://asreview.nl/ (latest accessed on 24 September 2024). Data from simulation studies show that the tool helps to detect 95% of relevant studies after screening between 8–33% of the studies originally detected (van de Schoot et al. 2021). Specifically, we loaded the titles and abstracts of the records identified by our search strategy for these two databases into the AS Review system. To train the algorithm to identify relevant studies, the first author categorized several records as either relevant or not (based on titles and abstracts, based on our inclusion criteria). Based on this initial categorization, the AS Review algorithm then classifies the remaining records as relevant or not. Then, the records identified as relevant are put forth by the algorithm for a person to judge as (ir)relevant. The algorithm is continually fine-tuned through the feedback provided on each judgment, such that the algorithm becomes more effective at distinguishing between relevant and irrelevant records. This leads to a growing number of relevant records at the beginning of the process that level out afterward. In our case, we conducted two separate screening processes, one for records from the WOS database and another for records from ProQuest. For the 6899 records identified in WOS, a total of 1149 records (17%) were manually reviewed and categorized by the first author, which led to the identification of 179 potentially relevant studies. For the 7659 records identified in the ProQuest database, 664 records (9%) were manually reviewed and categorized by the first author, which led to the identification of 58 potentially relevant dissertations. The full texts for these potentially relevant studies were retrieved and further assessed for eligibility.

In total, across our four search strategies, we identified 389 records as potentially relevant based on their titles and abstracts (cf. Figure 1). These were screened in-depth based on our inclusion criteria. After the exclusion of double records or studies that did not meet our criteria, our final dataset included 52 different samples stemming from 44 different studies (published between 1985 and 2023).

### 3.2. Coding Procedure

From the 52 samples fulfilling our inclusion criteria, one author extracted the correlation coefficients between GRA and cognitive ability, and these correlations were independently verified by at least one other author. We coded indicators from GRA and cognitive ability assessments such that higher values represent better GRA performance or higher cognitive ability scores (e.g., correlations based on reaction times or error rates were reverse-recoded).

To test our moderator hypotheses, we coded each effect size for a set of binary moderators. To examine whether measurement scope moderates the relationship between GRA and cognitive ability, we coded whether cognitive ability was measured using one vs. multiple (sub)tests (H2A) and whether one vs. more games were used (H2B). To examine whether the measurement medium moderates the relationship (H3), we coded whether the cognitive ability measure was a computer-based assessment (e.g., a working memory task executed on a computer or a computerized version of a traditional test) or a paper-pencil test. If no explicit description was given, and the original test source cited did not specify that a computerized medium of assessment was used, then we coded the assessment of cognitive ability as a paper-pencil test. Specifically, we coded the assessment as “paper-pencil” for 10 studies that did not explicitly state the medium of assessment (six of these studies used the Ravens Standard or Advanced Progressive Matrices, and four used the WAIS).

We further coded each effect size for the following binary moderators. To examine whether the type of cognitive ability measured moderates the relationship (RQ1), we coded whether the traditional cognitive ability measure aimed at assessing GMA (i.e., at Stratum III of the CHC model; e.g., Wonderlic test or ICAR) or a narrower cognitive ability (i.e., at Stratum II or I of the CHC model, such as processing speed). To examine whether the type of GRA employed moderates the relationship, we coded whether the GRA was based on an existing game (e.g., commercially available video game) and used as it is or if the GRA was specifically developed or adapted (e.g., for the assessment of specific constructs, or training purposes; RQ2A). Finally, to examine whether the purpose of the GRA moderates the relationship, we coded if the GRA was intended to specifically assess cognitive ability vs. other purposes (e.g., with the purpose to assess an alternative construct, like personality traits, or another purpose, such as training or playing; RQ2B).

### 3.3. Meta-Analytic Procedure

The meta-analysis was carried out using the metafor package in R version 4.3.3 (Viechtbauer 2010), which provides *p*-values (based on permutation test) and 95% confidence intervals. To compare data and stabilize sampling error variance (Viechtbauer 2010), we transformed the correlations to Fisher’s z metric (Borenstein et al. 2009), conducted all the analyses based on the *z* values, and then retransformed the results back into the *r* metric. The results presented below report the average correlations (i.e., *r*) and corresponding upper and lower limits. The 95% confidence intervals, which are symmetric in the z-metric, can become non-symmetric in the r-metric due to a maximum value of |1.00| for correlations.

As most of the studies identified by our search included more than one effect size, we applied multi-level meta-analytic procedures to account for the dependencies in our dataset. We adapted the outline by Assink and Wibbelink (2016) for a three-level meta-analysis, taking into account that effect sizes vary between participants (level 1), outcomes (level 2), and studies (level 3). We carried out the meta-analysis based on observed correlations extracted from the identified studies.

As we were interested in the overlap of the constructs assessed by GRAs and traditional cognitive ability tests, we also reported the results based on corrections for unreliability. We corrected correlations before transforming them to Fisher’s z values using the attenuation formula (Schmidt and Hunter 2015), which included the reliabilities of GRA and cognitive ability. Reliability coefficients were obtained from the original study where possible. We included different types of reliability estimates, including KR-20, intraclass correlation, and alternate form reliability. If a study reported multiple types of reliability estimates, we used the internal consistency reliability if it was reported, or else the test-retest reliability. In sum, 235 effect sizes for GRA and 174 effect sizes for cognitive ability could be corrected based on reliability values reported in the original study. For studies that did not report a reliability estimate, we corrected the observed correlation using the unweighted mean reliability values from our dataset. These were 0.71 for GRA and 0.74 for cognitive ability.

Subsequently, we conducted a series of separate analyses to test for each potential moderating effect stated in H2 and H3 and the two Research Questions. Additionally, to address the potential issue of multicollinearity of related moderators, we conducted a robustness check (Assink and Wibbelink 2016; Hox 2010). This robustness check involved conducting an omnibus test on a model that included all the moderators that were significant in the separate analyses.

## 4. Results

### 4.1. Study Characteristics

Our search and screening led to the identification of 807 relevant effect sizes stemming from 44 papers (cf. list of included papers in Appendix A) including 52 independent samples. Most of the studies were published in scientific journals. Only two studies were conference proceedings, and three were unpublished theses.

In total, our dataset is based on a maximum of *N* = 6139 participants. The sample size in the primary studies ranged from 12 to 633 participants, with 118.06 participants on average (SD = 142.43; median = 62) in the studies. Most samples were from the US (*m* = 20), followed by Spain (*m* = 7). There were three samples each from the UK, Australia, Germany, and Switzerland, and one sample each from Canada, Greece, Italy, Japan, and the Netherlands. The country of sampling was not explicitly mentioned in eight studies. Of the 52 samples that were included in this meta-analysis, 24 samples (46.15%) consisted exclusively of university students, five samples encompassed professional video gamers (9.62%), and three samples (5.77%) consisted of working populations. The remaining 20 samples (38.46%) comprised non-specified or mixed samples. A majority of the samples (*m* = 36; 69.23%) were collected in laboratory settings. Nine samples were collected with online studies or online experiments (17.31%), and insufficient information was reported in the remaining seven studies to report on their sample characteristics (13.46%). The mean age of participants in the samples ranged from 18.60 years (Buford and O’Leary 2015) to 70.67 years (Krebs et al. 2021), with an overall mean of 28.20 years across all included studies. We identified samples that consisted exclusively of female participants (Quiroga et al. 2011) or male participants (Jones et al. 1986; Rabbitt et al. 1989). Across all samples with information about gender composition, 50.86% of participants identified as female.

Nearly a third (*m* = 17; 32.69%) of the studies investigated the relationship between GRA and cognitive ability by using playful games (e.g., Tetris, League of Legends, Counterstrike). The remaining studies are based on either gamified assessments, gamefully designed assessments, or game-based assessments. These range from gamified VR-based versions of traditional cognitive ability tests (Borghetti et al. 2023) to specifically developed theory-driven assessments in the game environment of Minecraft (Ohlms et al. 2024).

### 4.2. Overall Relationships Between GRA and Cognitive Ability

**Figure 2:**
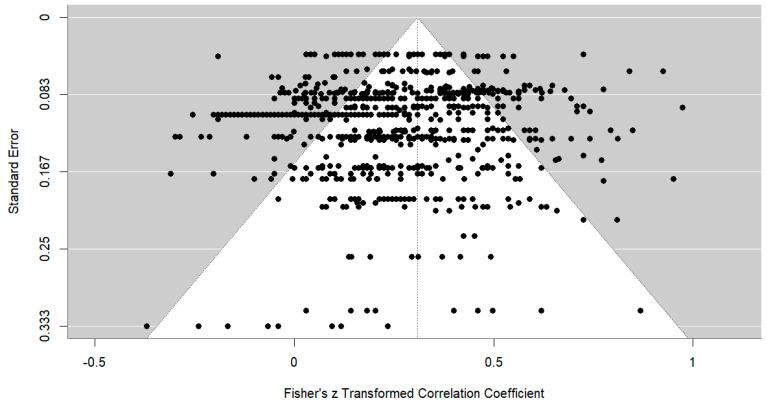
Funnel plot of observed correlations transformed into Fisher’s z.

The observed correlations included in this meta-analysis ranged from −0.35 up to +0.75 (cf. funnel plot with Fisher z transformed effect sizes and corresponding standard errors in Figure 2).

Modeling the effect sizes in a three-level model provided a better fit to the data than either of the two alternative models we examined: a model without within-study variance and a model without between-study variance (log-likelihood ratio test, *p* < .001). These results indicate significant variability of effect sizes between and within studies. In the three-level meta-analytic model, 20.25% of the variation occurred at level 1 (sampling variance), 39.82% at level 2 (within-study variance), and 39.93% at level 3 (between-study variance). Overall, the effect sizes in our dataset were heterogenous, *Q_E_*(806) = 3871.42; *p* < .001.

The estimated meta-analytic relationships are displayed in Table 2. We found support for our first hypothesis, with the overall observed mean correlation of GRA and traditional measures of cognitive ability to be *r* = 0.30, 95% CI [0.26; 0.34]. We also conducted all analyses with the corrected correlations, and these results are reported in Appendix B, Table A1.

### 4.3. Moderator Analysis

Schmidt and Hunter (2015) suggested that moderator analyses should be performed when less than 75% of the variance is at level 1. Therefore, we performed these analyses for H2–3 and reported the results in Table 2.

Supporting H2A, we found a significant moderating effect of measurement scope of cognitive ability, *Q_M_*(1) = 21.99; *p* < .001. We found a higher relationship between GRA and cognitive ability when the effect size was based on multiple ability tests (*r* = 0.39) than when it was based on a single test (*r* = 0.28). Supporting H2B, we found a significant moderating effect of measurement scope of GRA, *Q_M_*(1) = 4.23; *p* = .04. The relationship was higher when multiple performance indicators from GRAs were used (*r* = 0.38) than when a single GRA indicator was used (*r* = 0.29). However, most effect sizes in our dataset were based on single measures of GRA. Contrary to H3, we did not find a moderating effect for the administration medium of cognitive ability, *Q_M_*(1) = 3.09; *p* = .08. The relationship between GRA and traditional measures of cognitive ability was not significantly different when comparing effect sizes based on computer-based vs. paper-pencil assessment of cognitive ability. After conducting the robustness check, only the moderating effect of measurement scope for GMAs remained statistically significant, t(804) = 4.30, *p* < .001 (H2A).

We conducted three additional analyses to address our research questions. In response to RQ1, we found no significant moderating effect of effect sizes aimed at general vs. other levels of cognitive ability in the CHC model; *Q_M_*(1) = 0.16; *p* = .69. In response to RQ2, we also found no differences in the strength of the relationships comparing the relationship between GRA and cognitive ability for existing vs. specifically developed or adapted games; *Q_M_*(1) = 0.78; *p* = .38. Furthermore, we also found no differences in the relationship when GRAs were used with the explicit purpose to assess cognitive ability vs. other purposes; *Q_M_*(1) = 2.71; *p* = .10.

### 4.4. Additional Analysis

It seemed surprising (to us and the reviewers) that the explicit aim to assess cognitive ability did not moderate the relationship between GRAs and cognitive ability. However, it has to be noted that we simply coded for whether the game intended to measure cognitive ability (or not). Although some studies (e.g., Landers et al. 2021; Ohlms et al. 2024) used GRAs where the games were developed in a theory-driven way to assess specific cognitive abilities, not all games in this category based on our coding were developed in this fashion. For many of these games, it was unspecified and theoretically unclear how the game tasks connected to cognitive ability. Following the suggestion of one reviewer, we re-ran all our moderator analyses, but only in this particular subset of studies where GRAs intended to assess cognitive ability. We found three significant moderating effects, two of which remained significant in light of a robustness check.

First, we again found support for H2A about the measurement scope of cognitive ability; *Q_M_*(1) = 18.13; *p* < .001. There was a higher relationship between GRA and cognitive ability when the effect size was based on multiple cognitive ability tests (*r* = 0.41) than when it was based on a single test (*r* = 0.30). Second, in contrast to our results based on the full dataset, we found support for H3, about the measurement medium of cognitive ability; *Q_M_*(1) = 16.22; *p* < .001. In this subset of data, we found a higher relationship between GRA and cognitive ability when the effect sizes were based on computer-administered assessments of cognitive ability (*k* = 136, *m* = 10, *r* = 0.38) compared to paper-pencil tests (*k* = 222, *m* = 15, *r* = 0.29).

In addition, we checked for potential publication bias in our full dataset. The result of the test of an additional moderator coding for unpublished (n = 5; dissertations, thesis, or conference proceedings) vs. published studies was nonsignificant; *Q_M_*(1) = 1.20; *p* = .16.

## 5. Discussion

Our meta-analysis aimed to provide evidence on the relationship between a broad range of GRAs and traditional measures of cognitive ability. Building upon 807 correlation coefficients with a wide range of values extracted from the primary studies, we found a mean meta-analytic correlation of *r* = 0.30 between GRAs and traditional cognitive ability measures across 52 samples, including over 6000 adults. In addition, we found support for the idea that the relationship differs depending on the measurement scope, with the relationship between GRAs and cognitive ability being stronger when multiple ability tests are used. However, we found no convincing support that the relationship depends on the measurement scope of the games or the measurement medium of cognitive ability (paper-pencil vs. computerized assessment). Nonetheless, by uncovering shared and non-shared variance of GRA and traditional measures of cognitive ability, our results help to distinguish between the methods used to assess constructs and the constructs of interest itself.

Overall, we found that the relationship between GRA and traditional measures of cognitive ability on a general level is meaningful and positive. Assuming that GRAs and traditional cognitive ability measures tap into comparable demands and, at least partly, the same cognitive processes (Roman et al. 2024; van Lill et al. 2023), cognitive ability seems to be reflected in scores stemming from a broad range of GRA. As such, our finding indicates that there might be value in such modern approaches, opening up the possibility of measuring cognitive ability using assessments beyond standardized tests (Koch et al. 2021; Lievens and Reeve 2015). However, the obtained value of 0.30 is substantially lower in magnitude than values obtained in equivalence testing for cognitive ability, depending on the medium of administration (Mead and Drasgow 1993). Although correcting for unreliability results in higher estimates (with values of a maximum of 0.56), such values would not indicate identical constructs. So, although performance in GRA and scores stemming from traditional cognitive ability tests are correlated, they also seem to cover distinct constructs.

We also found heterogeneous relationships in the primary studies identified in the literature. The correlations of indicators stemming from GRAs and traditional cognitive ability measures ranged from moderately negative to strongly positive, suggesting that some GRA might be better at assessing cognitive ability than others. In terms of potential moderators, we found in our study evidence that using multiple indicators of cognitive ability enhances validity, as expected. However, in our overall dataset, no other moderating effects were supported. The assessment scope of the game and the medium used to assess cognitive ability did not moderate the relationship. Further, the strength of the relationship did not depend on whether general vs. more specific ability was assessed. On the GRA side, the strength of the relationships with traditional cognitive ability measures did not depend on the purpose of the GRA, whether it was an existing game or one developed specifically to assess cognitive ability. It was only in the subset of studies where GRAs aimed to assess cognitive ability that the medium used to assess cognitive ability moderated the relationship. We found a stronger relationship when cognitive ability was assessed using a computer compared to traditional paper-pencil tests.

However, we identify only a handful of studies where GRAs were intended specifically to assess cognitive ability and even fewer studies that used a theory-driven approach to develop such GRAs (e.g., Landers et al. 2021; Ohlms et al. 2024). Therefore, future research should investigate which specific abilities within the CHC model are assessed by different types of GRAs. Furthermore, we do not know which specific gaming features might strengthen the relationship between GRAs and cognitive ability. For example, Quiroga et al. (2009) suggested that computer games should have medium complexity, low consistency across items, and avoid relying on transfer keys to achieve high overlap with cognitive ability. We recommend that future research systematically investigate these potentially moderating factors and closely examine the different game mechanics outlined by Hervas et al. (2017) to determine which games are most effective at targeting specific cognitive abilities.

Given the overall effect size, even after correcting for unreliability, can GRAs be used as a valid assessment tool for measuring cognitive ability? There are several potential reasons why the overlap between GRAs and traditional cognitive ability measures is not higher. On a theoretical level, GRAs and traditional tests may emphasize different ability constructs or different aspects of the same ability construct. For example, games often require visual processing and short-term memory, whereas paper-pencil tests tend to assess reading, writing, and quantitative knowledge. If there is a low correspondence between the cognitive abilities targeted by each method, the observed correlation will naturally be lower, a concept explained by Brunswik’s (1955) lens model. Indeed, higher relationships are expected when GRAs and cognitive ability tests assess the same constructs. However, we were not always able to match the underlying constructs, as our data set also included commercial games not specifically designed to assess cognitive ability. Along with exploratory research in this area, investigating the relationship between GRAs and traditional cognitive ability tests without explicitly stating which kind of connections or overlap was expected, made it difficult to specify which cognitive abilities were targeted by the particular GRA.

Additionally, game performance may require other constructs beyond cognitive ability, such as game-specific skills or motivational traits, which can diminish the role of cognitive ability on GRA performance. Errors specific to GRAs, such as accessibility issues, familiarity with the game, or the type of feedback provided (Hawkes et al. 2018), might also systematically affect performance in GRAs. Together, these differences might lead to varying relationships with traditional cognitive ability tests.

Finally, as expected, correcting for unreliability results in higher estimates, which is often taken to reflect the “true correlation” between GRA and cognitive ability. However, the specific correction method could have impacted our results. While some researchers have made strong arguments for correcting meta-analytic values (Schmidt and Hunter 2015), it is also possible to overcorrect (Sackett et al. 2022). In our case, reliability corrections led to correlations above 1.0 in seven cases, which required that we either excluded these cases or used alternative estimates. In each of these cases, low reliabilities were reported (e.g., 0.16), raising further questions about how to adequately assess reliability in GRAs. For example, the problem of suitable reliability estimates emerges when confronted with scores in diverse mini-games, performance across different gaming cycles, or practice effects when playing a particular game for several weeks (e.g., Quiroga et al. 2011).

Our findings have several theoretical implications. We showed that the relationship between GRA and cognitive ability generalizes across different adult samples. By shedding light on the shared and non-shared variance of GRA and traditional cognitive ability tests, our findings indicate similarities but also substantial differences. As such, our results pave the way for theoretical advancements to understand performance in GRA and the assessment of cognitive ability beyond traditional tests (Koch et al. 2021). Furthermore, our results have implications for using GRAs in practice. While indicators from GRAs overlap with cognitive ability tests, the magnitude of the relationship suggests that they are not equivalent measures. However, our findings, which show no significant moderating effects on the GRA side, suggest that different GRAs could be used in practice to assess cognitive ability. Since the purpose of the GRA does not seem to impact the relationship, one could infer a player’s cognitive ability from a wide range of games. That said, more research is needed on theory-driven games that are designed to assess specific cognitive abilities and processes.

Despite the contribution of our study to the research field, we acknowledge certain limitations. First, alternative methodological choices in the meta-analysis might have led to different results. As we based our analysis on zero-order correlations, a higher relationship between performance indicators from GRAs and measures of cognitive ability might be obtained by modeling the relationship on the latent level (e.g., Landers et al. 2021). Also, executing other corrections (e.g., range restriction) would have affected the results reported here. Furthermore, some of the moderators we examined may have been difficult to detect due to the limited representation of certain categories in our dataset (e.g., very few unpublished studies and relatively few studies using GRA to assess cognitive ability). More high-quality primary research is needed to test these and other potential moderating factors of the relationship between GRAs and traditional measures of cognitive ability. Second, our meta-analytic findings are primarily based on published papers. We identified only five unpublished studies: two from conference proceedings and three unpublished theses. Although we contacted authors of potentially relevant studies, none of the 20 authors contacted replied with sufficient statistical information to include their studies in the analysis. While focusing on published studies ensures the rigor of the research included in this review, it could lead to an overestimation of the real relationship due to publication bias. However, several factors suggest that our database is representative of research in this field. First, the effect sizes in our dataset were heterogeneous (ranging from values of −0.30 up to +0.75; cf. funnel plot in Figure 2). Second, the correlations were based on both hypothesis-driven and exploratory studies, which included a fair number of non-significant findings. Third, our choice to focus on the observed—rather than corrected—correlations suggests that overestimation is less likely to have occurred in this instance. There might also be several commercially available solutions for GRAs that effectively assess cognitive ability (Georgiou et al. 2019; Ohlms et al. 2024). Including such data could have led to higher estimates of the relationship between GRA and cognitive ability. However, we did not include these, given that such information is often restricted due to intellectual property protections and client confidentiality. Therefore, future research is strongly encouraged to attempt to expand the overall database by, for example, contacting test publishers, commercial video game companies, practitioners, or government agencies to obtain additional reports of relevant research.

While we identified an overall positive relationship between GRAs and traditional cognitive ability tests, future research is needed to explore this relationship. For example, future studies should investigate how different GRAs relate to specific cognitive abilities based on the abilities outlined in the CHC model (McGrew 2009). Additionally, the distinction between speed and power in traditional cognitive ability measures (Mead and Drasgow 1993) could help explain variations in the relationship. To extend our knowledge about the nature of cognitive abilities, an interdisciplinary approach drawing from computer science, neuroscience, and biology (Koch et al. 2021), may also provide deeper insights into how assessment methods relate to underlying cognitive abilities. On the side of GRAs, the game features and design decisions that influence GRA performance need to be clarified (Ohlms et al. 2024). Besides general features identified in video games (Quiroga et al. 2009), it is crucial to investigate how specific features in game-based assessments affect its construct validity and impact the effectiveness of GRAs in terms of measuring cognitive abilities. For example, elements such as the level of playfulness, the use of virtual reality or avatars, and differences in game genres, especially in terms of storytelling, need to be explored (e.g., Ohlms et al. 2024).

Furthermore, not all studies in our meta-analysis fully utilized the rich data available from GRAs, which could offer new ways to target cognitive ability by analyzing process-oriented, behavioral trace data with machine learning (ML) algorithms (Auer et al. 2022). Using more detailed information from the gaming situation—rather than static indicators like an overall score (e.g., the number of problems solved in a certain time frame)—enables the use of multiple, process-oriented measures. When combined with the aforementioned ML approaches, these may provide a more accurate assessment and prediction of cognitive ability (Koch et al. 2021).

Future research should also take a modular approach to explore how different GRA features impact their validity and applicant reactions, especially in personnel selection contexts (Lievens and Sackett 2017). While it is often assumed that gamification results in positive reactions from applicants (e.g., Georgiou et al. 2019), some studies have reported less favorable reactions to game-based assessment compared to paper-pencil tests (Ohlms et al. 2024). There is also an urgent need to investigate the predictive validity of GRAs for academic and work performance. To our knowledge, only a few studies have examined the validity of GRAs for predicting grades in school or university settings (e.g., Hommel et al. 2022; Malanchini et al. 2021) or performance in work settings (e.g., Landers et al. 2021; Melchers and Basch 2022). For example, Landers et al. (2021) reported correlations of *r* = 0.29 between their game-based assessment targeting general mental ability and supervisor ratings of job performance. However, it remains unclear whether GRAs provide incremental predictive value for academic and work outcomes beyond traditional selection instruments. For example, Melchers and Basch (2022) reported a substantial correlation of *r* = 0.18 between performance in a computer-based simulation game (running a fictitious organization) and performance stemming from eight exercises from an assessment center. Lastly, considering the importance of equity and fairness in modern assessment methods (Holden and Tanenbaum 2023), more research is needed to identify potential confounding factors or biases in using GRAs. Prior research findings have already highlighted that age, gender, and prior gaming experience can influence performance (e.g., Foroughi et al. 2016; Landers et al. 2021; Melchers and Basch 2022). Therefore, those looking to use GRAs in practice must ensure that GRAs produce psychometrically unbiased outcomes for different applicant groups across different cultural contexts.

## 6. Conclusions

We provide meta-analytic evidence for a robust (albeit moderate) positive correlation between indicators stemming from GRA and traditional measures of cognitive ability, which might represent a common shared core of the underlying construct. Furthermore, we demonstrate that the relationship is stronger when multiple cognitive ability measures are used. Interestingly, GRAs specifically developed to assess cognitive ability are not more strongly related to traditional measures of cognitive ability than GRAs with other purposes. Therefore, future research is urged to take a closer look at the specific gaming mechanics that might enhance or limit the use of GRAs in practice to assess different aspects of cognitive ability.

## Figures and Tables

**Table 1:** Inclusion criteria meta-analysis (and examples for types of excluded effect sizes).

Criteria	Description & Examples
1. Study design & statistics	Empirical study: reporting results of quantitative data collection; correlations between GRA and traditional cognitive ability measure. Examples of exclusion: meta-analysis, overview, or review articles; no correlation available (even upon request)
2. GRA	Limited to technology-based GRAs. GRAs had to (i) include at least one game mechanism identified by Hervas et al. (2017) and (ii) report an objective individual performance measure unaffected by others (e.g., game score or rank). Examples of exclusion: Non-digital games (e.g., board games); no game mechanics applied (only game-framing used); relied solely on subjective gaming indicators (e.g., self-reported experience); games depended on the performance of others (e.g., social exchange games)
3. Cognitive ability	Traditional assessment of cognitive ability can be located in the CHC model, including paper-pencil and computer-based assessment. Examples of exclusion: other measures used, e.g., EEG indicators, or measures to diagnose impairment of cognitive functioning (e.g., screening for signs of dementia), or assessment of solely personality traits
4. Sample	Sample participants had to be healthy adults (aged 18 or older); the study had to report sample size. Examples of exclusion: samples with participants younger than 18 years (e.g., school children); clinical samples (e.g., dementia patients, care settings); not enough information to judge the sample based on our criteria
Other criteria	Published studies in scientific journals, papers available in conference proceedings, or university repositories. Years: all available until December 2023. Language: German, English, or Dutch.

**Table 2:** Results of meta-analyses on the relationship between game-related assessment (GRA) and cognitive ability (based on observed correlations).

	*k*	*N*	*m*	r_obs_	95%CI	
					LB	UB
H1: Overall relationship	807	6139	52	0.30 ***	0.26	0.34
** Estimates based on a set of binary moderators according to hypothesis**						
H2A: Measurement scope cognitive ability						
Yes (multiple tests)	102	4451	17	0.39 ***	0.33	0.44
No (single test)	705	2521	43	0.28 ***	0.24	0.32
H2B: Measurement scope GRA						
Yes (multiple games)	38	1678	7	0.38 ***	0.26	0.48
No (single game)	769	5026	46	0.29 ***	0.25	0.33
H3: Computer-based measurement of cognitive ability						
Yes (computer-based)	363	2544	25	0.30 ***	0.24	0.35
No (paper-pencil)	444	4095	34	0.31 ***	0.27	0.36
**Estimates based on a set of binary moderators according to research questions**						
RQ1: General cognitive ability/GMA measured in traditional test						
Yes (g/GMA)	27	1033	8	0.30 ***	0.21	0.38
No (broad or narrow abilities)	780	5287	46	0.30 ***	0.26	0.34
RQ2.A: Existing GRA						
Yes	516	2288	30	0.27 ***	0.23	0.34
No (specifically developed/adapted)	291	3851	22	0.31 ***	0.27	0.36
RQ2.B: GRA assessment of cognitive ability						
Yes (intend to assess cognitive ability)	358	3946	21	0.32 ***	0.29	0.36
No (other purposes)	449	2193	31	0.28 ***	0.22	0.33

*Notes. k* = number of coefficients; *N* = total number of participants (max); *m* = number of independent samples; r_obs_ = mean observed correlation. CI = confidence interval for correlation coefficients; LL = lower level; UL = upper level. *** *p* < .001.

## Data Availability

The raw data set is openly available at the OSF: https://osf.io/2k6hz/?view_only=2144f80a5c1e4c399ad7a4acbfdffa04 (accessed on 10 December 2024).

## Note

1 It is important to distinguish GRAs from work simulations, as they can easily be conflated. Work simulations are contextualized assessment procedures designed to mimic key psychological and physical aspects of a job (Lievens and De Soete 2012). Both simulations and GRAs aim to assess job-relevant skills, but they differ in intent. GRAs are designed with playfulness in mind, using game mechanics to motivate and engage users. In contrast, work simulations are designed to replicate real-world work scenarios and experiences. In work simulations, tasks must closely resemble actual work behaviors (e.g., an air traffic control simulation involves directing aircrafts to prevent flight conflicts). In contrast, GRAs do not need to resemble work scenarios. Their gameplay might involve job-related, but also unrelated activities such as bursting balloons or catching fish. The emphasis in GRAs is on using game mechanics to create an engaging experience rather than mimicking specific job tasks. Although a GRA can include simulation elements (e.g., through the game mechanics of goals or immersion), simulation is not inherently part of a GRA (Landers and Sanchez 2022). Conversely, a work simulation that merely replicates job tasks (e.g., reading emails) without incorporating game mechanics that encourage deep immersion in the game play (e.g., story narratives and specified roles), would not constitute a GRA. In summary, while GRAs and work simulations overlap, the primary differentiator is the intentional use of game mechanics and the element of playfulness in GRAs, as opposed to the replication of job-related scenarios in work simulations that do not need to involve game mechanics.

## Appendix A. Studies Used in Meta-Analysis (44 Papers)

1. Aalbers, T., M. A. Baars, M. G. Olde Rikkert, and R. P. Kessels. 2013. “Puzzling with online games (BAM-COG): reliability, validity, and feasibility of an online self-monitor for cognitive performance in aging adults.” *J Med Internet Res* 15 (12):e270. https://doi.org/10.2196/jmir.2860.
2. Adams, D., and R. Mayer. 2012. “Examining the Connection Between Dynamic and Static Spatial Skills and Video Game Performance.” Proceedings of the Annual Meeting of the Cognitive Science Society.
3. Atkins, S. M., A. M. Sprenger, G. J. H. Colflesh, T. L. Briner, J. B. Buchanan, S. E. Chavis, S. Chen, G. L. Iannuzzi, V. Kashtelyan, E. Dowling, J. I. Harbison, D. J. Bolger, M. F. Bunting, and M. R. Dougherty. 2014. “Measuring Working Memory Is All Fun and Games.” *Experimental Psychology* 61 (6):417–438. https://doi.org/10.1027/1618-3169/a000262.
4. Auer, E. M., G. Mersy, S. Marin, J. Blaik, and R. N. Landers. 2022. “Using machine learning to model trace behavioral data from a game-based assessment.” *International Journal of Selection and Assessment* 30 (1):82–102. https://doi.org/10.1111/ijsa.12363.
5. Baniqued, P. L., H. Lee, M. W. Voss, C. Basak, J. D. Cosman, S. DeSouza, J. Severson, T. A. Salthouse, and A. F. Kramer. 2013. “Selling points: What cognitive abilities are tapped by casual video games?” *Acta Psychologica* 142 (1):74–86. https://doi.org/10.1016/j.actpsy.2012.11.009.
6. Bonny, J. W., and L. M. Castaneda. 2017. “Number processing ability is connected to longitudinal changes in multiplayer online battle arena skill.” *Computers in Human Behavior* 66:377–387. https://doi.org/10.1016/j.chb.2016.10.005.
7. Bonny, J. W., L. M. Castaneda, and T. Swanson. 2016. “Using an International Gaming Tournament to Study Individual Differences in MOBA Expertise and Cognitive Skills.” Proceedings of the 2016 CHI Conference on Human Factors in Computing Systems, San Jose, California, USA.
8. Borghetti, D., C. Zanobini, I. Natola, S. Ottino, A. Parenti, V. Brugada-Ramentol, H. Jalali, and A. Bozorgzadeh. 2023. “Evaluating cognitive performance using virtual reality gamified exercises.” *Frontiers in Virtual Reality* 4. https://doi.org/10.3389/frvir.2023.1153145.
9. Buford, C. Colby, and B. J. O’Leary. 2015. “Assessment of Fluid Intelligence Utilizing a Computer Simulated Game.” *International Journal of Gaming and Computer-Mediated Simulations* 7 (4):1–17. https://doi.org/10.4018/ijgcms.2015100101.
10. Cheng, X., G. C. Gilmore, A. J. Lerner, and K. Lee. 2023. “Computerized Block Games for Automated Cognitive Assessment: Development and Evaluation Study.” *JMIR Serious Games* 11:e40931. https://doi.org/10.2196/40931.
11. Chesham, A., S. M. Gerber, N. Schutz, H. Saner, K. Gutbrod, R. M. Muri, T. Nef, and P. Urwyler. 2019. “Search and Match Task: Development of a Taskified Match-3 Puzzle Game to Assess and Practice Visual Search.” *JMIR Serious Games* 7 (2):e13620. https://doi.org/10.2196/13620.
12. Chicchi Giglioli, I. A., C. de Juan Ripoll, E. Parra, and M. Alcañiz Raya. 2018. “EXPANSE: A novel narrative serious game for the behavioral assessment of cognitive abilities.” *PLOS ONE* 13 (11):e0206925. https://doi.org/10.1371/journal.pone.0206925.
13. Cretenoud, A. F., A. Barakat, A. Milliet, O. H. Choung, M. Bertamini, C. Constantin, and M. H. Herzog. 2021. “How do visual skills relate to action video game performance?” *J Vis* 21 (7):10. https://doi.org/10.1167/jov.21.7.10.
14. Denga Ndemera, R. A. 2022. “Effects of Playing the Video Game Tetris on Attention and Processing Speed.” 29254213 Ph.D., Rensselaer Polytechnic Institute.
15. Foroughi, C. K., C. Serraino, R. Parasuraman, and D. A. Boehm-Davis. 2016. “Can we create a measure of fluid intelligence using Puzzle Creator within Portal 2?” *Intelligence* 56:58–64. https://doi.org/10.1016/j.intell.2016.02.011.
16. Gagnon, D. 1985. “Videogames and spatial skills: An exploratory study.” *Ectj* 33 (4):263–275.
17. Gödöllei Lappalainen, F. A. 2017. “Game-Based Assessments of Cognitive Ability: Validity and Effects on Adverse Impact through Perceived Stereotype Threat, Test-Taking Motivation and Anxiety.” Master thesis University of Calgary.
18. Jones, M. B., W. P. Dunlap, and I. M. Bilodeau. 1986. “Comparison of video game and conventional test performance.” *Simulation & Games* 17 (4):435–446.
19. Kokkinakis, A. V., P. I. Cowling, A. Drachen, and A. R. Wade. 2017. “Exploring the relationship between video game expertise and fluid intelligence.” *PLOS ONE* 12 (11):e0186621. https://doi.org/10.1371/journal.pone.0186621.
20. Kranz, M. B., P. L. Baniqued, M. W. Voss, H. Lee, and A. F. Kramer. 2017. “Examining the Roles of Reasoning and Working Memory in Predicting Casual Game Performance across Extended Gameplay.” *Front Psychol* 8:203. https://doi.org/10.3389/fpsyg.2017.00203.
21. Krebs, C., M. Falkner, J. Niklaus, L. Persello, S. Klöppel, T. Nef, and P. Urwyler. 2021. “Application of Eye Tracking in Puzzle Games for Adjunct Cognitive Markers: Pilot Observational Study in Older Adults.” *JMIR Serious Games* 9 (1):e24151. https://doi.org/10.2196/24151.
22. Kröner, S., and D. Leutner. 2022. “MultiFlux–Pilotstudie für die Entwicklung eines Verfahrens zur simulationsbasierten Intelligenzdiagnostik [MultiFlux—A pilot study concerning the development of a stimulation-based tool for intelligence assessment].” *Zeitschrift fur Arbeits und Organisationspsychologie* 46: 84–88.
23. Landers, R. N., M. B. Armstrong, A. B. Collmus, S. Mujcic, and J. T. Blaik. 2021. “Theory-driven game-based assessment of general cognitive ability: Design theory, measurement, prediction of performance, and test fairness.” *Journal of Applied Psychology*. https://doi.org/10.1037/apl0000954.
24. Lee, K., D. Jeong, R. C. Schindler, and E. J. Short. 2016. “SIG-Blocks: Tangible game technology for automated cognitive assessment.” *Computers in Human Behavior* 65:163–175. https://doi.org/10.1016/j.chb.2016.08.023.
25. Leutner, F., S. C. Codreanu, S. Brink, and T. Bitsakis. 2022. “Game based assessments of cognitive ability in recruitment: Validity, fairness and test-taking experience.” *Front Psychol* 13:942662. https://doi.org/10.3389/fpsyg.2022.942662.
26. Lim, J., and A. Furnham. 2018. “Can Commercial Games Function as Intelligence Tests? A Pilot Study.” *The Computer Games Journal* 7 (1):27–37. https://doi.org/10.1007/s40869-018-0053-z.
27. Martin, N., J. Capman, A. Boyce, K. Morgan, M.F. Gonzalez, and S. Adler. 2020. “New frontiers in cognitive ability testing: working memory.” *Journal of Managerial Psychology* 35 (4):193–208. https://doi.org/https://doi.org/10.1108/JMP-09-2018-0422.
28. McPherson, J., and N. R. Burns. 2007. “Gs invaders: Assessing a computer game-like test of processing speed.” *Behavior research methods* 39:876–883.
29. McPherson, J., and N. R. Burns. 2008. “Assessing the validity of computer-game-like tests of processing speed and working memory.” *Behav Res Methods* 40 (4):969–81. https://doi.org/10.3758/BRM.40.4.969.
30. Nikolaou, I., K. Georgiou, and V. Kotsasarlidou. 2019. “Exploring the Relationship of a Gamified Assessment with Performance.” *Span J Psychol* 22:E6. https://doi.org/10.1017/sjp.2019.5.
31. Ohlms, M. L., K. G. Melchers, and U. P. Kanning. 2024. “Can we playfully measure cognitive ability? Construct-related validity and applicant reactions.” *International Journal of Selection and Assessment* 32:91–107. https://doi.org/10.1111/ijsa.12450.
32. Ono, T., T. Sakurai, S. Kasuno, and T. Murai. 2022. “Novel 3-D action video game mechanics reveal differentiable cognitive constructs in young players, but not in old.” *Sci Rep* 12 (1):11751. https://doi.org/10.1038/s41598-022-15679-5.
33. Pontrelli, M. James. 1990. “A study of the relationship between practice in the use of a radar simulation game and ability to negotiate spatial orientation problems.” 9119893 Ed.D., Oklahoma State University.
34. Quiroga, M. A., A. Diaz, F. J. Román, J. Privado, and R. Colom. 2019. “Intelligence and video games: Beyond “brain-games”.” *Intelligence* 75:85–94. https://doi.org/10.1016/j.intell.2019.05.001.
35. Quiroga, M. A., M. Herranz, M. Gómez-Abad, M. Kebir, J. Ruiz, and Roberto Colom. 2009. “Video-games: Do they require general intelligence?” *Computers & Education* 53 (2):414–418. https://doi.org/10.1016/j.compedu.2009.02.017.
36. Quiroga, M. Á., F. J. Román, A. Catalán, H. Rodríguez, J. Ruiz, M. Herranz, M. Gómez-Abad, and R. Colom. 2011. “Videogame performance (not always) requires intelligence “ *International Journal of Online Pedagogy and Course Design (IJOPCD),* 1 (3):18–32.
37. Quiroga, M. A., F. J. Román, J. De La Fuente, J. Privado, and R. Colom. 2016. “The measurement of intelligence in the XXI century using video games.” *The Spanish Journal of Psychology* 19: E89.
38. Rabbitt, P., N. Banerji, and A. Szymanski. 1989. “Space Fortress as an IQ test? Predictions of learning and of practised performance in a complex interactive video-game.” *Acta psychologica* 71(1–3) (1–3):243–257.
39. Roman, F. J., P. Gutierrez, J. Ramos-Cejudo, P. A. Gonzalez-Calero, P. P. Gomez-Martin, C. Larroy, R. Martin-Brufau, C. Lopez-Cavada, and M. A. Quiroga. 2024. “Checking Different Video Game Mechanics to Assess Cognitive Abilities in Groups with and without Emotional Problems.” *J Intell* 12 (1). https://doi.org/10.3390/jintelligence12010001.
40. Simons, A., I. Wohlgenannt, S. Zelt, M. Weinmann, J. Schneider, and J. vom Brocke. 2023. “Intelligence at play: game-based assessment using a virtual-reality application.” *Virtual Reality* 27 (3):1827–1843. https://doi.org/10.1007/s10055-023-00752-9.
41. Thompson, O., S. Barrett, C. Patterson, and D. Craig. 2012. “Examining the Neurocognitive Validity of Commercially Available, Smartphone-Based Puzzle Games.” *Psychology* 03 (07):525–526. https://doi.org/10.4236/psych.2012.37076.
42. Valls-Serrano, C., C. de Francisco, E. Caballero-López, and A. Caracuel. 2022. “Cognitive Flexibility and Decision Making Predicts Expertise in the MOBA Esport, League of Legends.” *SAGE Open* 12 (4):215824402211427. https://doi.org/10.1177/21582440221142728.
43. Ventura, M., V. Shute, T. Wright, and W. Zhao. 2013. “An investigation of the validity of the virtual spatial navigation assessment.” *Front Psychol* 4:852. https://doi.org/10.3389/fpsyg.2013.00852.
44. Wang, P., Y. Fang, J.-Y. Qi, and H.J. Li. 2023. “FISHERMAN: A Serious Game for Executive Function Assessment of Older Adults.” *Assessment* 30 (5):1499–1513. https://doi.org/10.1177/10731911221105648.

## Appendix B. Result Table with Correlations Corrected for Unreliability

**Table A1:** Meta-analytic relations between game-related assessment (GRA) and cognitive ability based on correlations corrected for unreliability in GRA and cognitive ability.

	*k*	*m*	r_cor_	95%CI	
				LB	UB
H1: Overall relationship	807	52	0.45 ***	0.38	0.52
Test of a set of binary moderators according to hypothesis					
H2A: Measurement scope cognitive ability					
Yes (multiple tests)	102	17	0.56 ***	0.48	0.63
No (single test)	705	43	0.43 ***	0.36	0.50
H2B: Measurement scope GRA					
Yes (multiple games)	38	7	0.54 ***	0.37	0.68
No (single game)	769	46	0.44 ***	0.37	0.51
H3: Computer-based measurement of cognitive ability					
Yes (computer-based)	363	25	0.46 ***	0.34	0.56
No (paper-pencil)	444	34	0.48 ***	0.40	0.56
Test of a set of binary moderators according to research questions					
RQ1: General cognitive ability/GMA measured in traditional test					
Yes (g/GMA)	27	8	0.39 ***	0.24	0.52
No (broad or narrow abilities)	780	46	0.46 ***	0.39	0.53
RQ2.A: Existing GRA					
Yes	516	30	0.40 ***	0.32	0.46
No (specifically developed or adapted)	291	22	0.52 ***	0.41	0.62
RQ2.B: GRA assessment of cognitive ability					
Yes (intend to assess cognitive ability)	358	21	0.49 ***	0.42	0.56
No (other purpose)	449	31	0.42 ***	0.31	0.51

*Notes. k* = number of coefficients; *m* = number of independent samples; r_cor_ = mean observed correlation corrected for unreliability in GRA and cognitive ability (unweighted). Values to correct for unreliability were extracted from the corresponding papers, or estimated with the mean unweighted average value of 0.71 (GRA) and 0.74 (cognitive ability). Given that the corrections led in seven cases to correlation coefficient estimates above a value of 1.0 (e.g., for studies with rather low reported reliability, with values of 0.16–0.20), these correlations were set to 0.99 before the analysis. Moderator analysis carried out led only to a significant effect for measurement scope of cognitive ability; *Q_M_*(1) = 6.45; *p* = .01. CI = confidence interval; LL = lower level; UL = upper level. *** *p* < .001.

## References

[B1-jintelligence-12-00129] Ackerman Paul L., Flanagan Dwan P., McDonough Erin M. (2012). Intelligendce-as-process, personality, interests, and intelligence-as-knowledge. Contemporary Intellectual Assessment: Theories, Tests, and Issues.

[B2-jintelligence-12-00129] Adams Deanne, Mayer Rich (2012). Examining the Connection Between Dynamic and Static Spatial Skills and Video Game Performance. Paper presented at the Annual Meeting of the Cognitive Science Society.

[B3-jintelligence-12-00129] Adler Seymour, Boyce Anthony S., Caputo Pat M., Scott John C., Bartram Dave, Reynolds Douglas H. (2018). Employment testing. Next Generation Technology-Enhanced Assessment.

[B4-jintelligence-12-00129] Arthur Winfred, Villado Anton J. (2008). The importance of distinguishing between constructs and methods when comparing predictors in personnel selection research and practice. Journal of Applied Psychology.

[B5-jintelligence-12-00129] Assink Mark, Wibbelink Carlijn J. M. (2016). Fitting three-level meta-analytic models in R: A step-by-step tutorial. The Quantitative Methods for Psychology.

[B6-jintelligence-12-00129] Atkins Sharona M., Sprenger Amber M., Colflesh Gregory J. H., Briner Timothy L., Buchanan Jacob B., Chavis Sydnee E., Chen Sy-yu, Iannuzzi Gregory L., Kashtelyan Vadim, Dowling Eamon (2014). Measuring Working Memory Is All Fun and Games. Experimental Psychology.

[B7-jintelligence-12-00129] Auer Elena M., Mersy Gabriel, Marin Sebastian, Blaik Jason, Landers R. N. (2022). Using machine learning to model trace behavioral data from a game-based assessment. International Journal of Selection and Assessment.

[B8-jintelligence-12-00129] Baniqued Pauline L., Lee Hyunkyu, Voss Michelle W., Basak Chandramallika, Cosman Joshua D., DeSouza Shanna, Severson Joan, Salthouse Timothy A., Kramer Arthur F. (2013). Selling points: What cognitive abilities are tapped by casual video games?. Acta Psychologica.

[B9-jintelligence-12-00129] Borenstein Michael, Hedges Larry V., Higgins Julian P. T., Rothstein Hannah R. (2009). Introduction to Meta-Analysis.

[B10-jintelligence-12-00129] Borghetti Davide, Zanobini Carlotta, Natola Ilenia, Ottino Saverio, Parenti Angela, Brugada-Ramentol Victòria, Jalali Hossein, Bozorgzadeh Amir (2023). Evaluating cognitive performance using virtual reality gamified exercises. Frontiers in Virtual Reality.

[B11-jintelligence-12-00129] Brunswik Egon (1955). Representative design and probabilistic theory in a functional psychology. Psychological Review.

[B12-jintelligence-12-00129] Buford Charles Colby, O’Leary Brian J. (2015). Assessment of Fluid Intelligence Utilizing a Computer Simulated Game. International Journal of Gaming and Computer-Mediated Simulations.

[B13-jintelligence-12-00129] Campbell Donald T., Fiske Donald W. (1959). Convergent and discriminant validation by the multitrait-multimethod matrix. Psychological Bulletin.

[B14-jintelligence-12-00129] Chicchi Giglioli Irene Alice, Ripoll Carla de Juan, Parra Elena, Raya Mariano Alcañiz (2018). EXPANSE: A novel narrative serious game for the behavioral assessment of cognitive abilities. PLoS ONE.

[B15-jintelligence-12-00129] Cretenoud Aline F., Barakat Arthur, Milliet Alain, Choung Oh-Hyeon, Bertamini Marco, Constantin Christophe, Herzog Michael H. (2021). How do visual skills relate to action video game performance?. Journal of Vision.

[B16-jintelligence-12-00129] Deterding Sebastian, Dixon Dan, Khaled Rilla, Nacke Lennart (2011). From game design elements to gamefulness: Defining “gamification”. Paper presented at the 15th International Academic MindTrek Conference: Envisioning Future Media Environments.

[B17-jintelligence-12-00129] Dilchert Stephen, Ones Deniz S., Anderson Neal, Viswesvaran Chockalingam, Sinangil Handan K. (2018). Cognitive ability. The SAGE Handbook of Industrial, Work & Organizational Psychology: Personnel Psychology and Employee Performance.

[B18-jintelligence-12-00129] Ellingsen Victor J., Engle Randall W., Sternberg Robert J. (2020). Cognitive approaches to intelligence. Intelligence.

[B19-jintelligence-12-00129] Fetzer Michael, McNamara Jennifer, Geimer Jennifer L. (2017). Gamification, Serious Games and Personnel Selection. The Wiley Blackwell Handbook of the Psychology of Recruitment, Selection and Employee Retention.

[B20-jintelligence-12-00129] Flanagan Dawn P., McDonough Erin M. (2018). Contemporary Intellectual Assessment.

[B21-jintelligence-12-00129] Foroughi Cyrus K., Serraino Carolyn, Parasuraman Raja, Boehm-Davis Deborah A. (2016). Can we create a measure of fluid intelligence using Puzzle Creator within Portal 2?. Intelligence.

[B22-jintelligence-12-00129] Georgiou Konstantina, Gouras Athanasios, Nikolaou Ioannis (2019). Gamification in employee selection: The development of a gamified assessment. International Journal of Selection and Assessment.

[B23-jintelligence-12-00129] Harman Jason L., Brown Kayla D. (2022). Illustrating a narrative: A test of game elements in game-like personality assessment. International Journal of Selection and Assessment.

[B24-jintelligence-12-00129] Hawkes Ben, Cek Iva, Handler Charles A., Scott John C., Bartram Dave, Reynolds Douglas H. (2018). The gamification of employee selection tools. Next Generation Technology-Enhanced Assessment.

[B25-jintelligence-12-00129] Hervas Ramon, Ruiz-Carrasco David, Mondejar Tania, Bravo Jose (2017). Gamification mechanics for behavioral change: A systematic review and proposed taxonomy. Paper presented at the 11th EAI International Conference on Pervasive Computing Technologies for Healthcare.

[B26-jintelligence-12-00129] Holden LaTasha R., Tanenbaum Gabriel J. (2023). Modern Assessments of Intelligence Must Be Fair and Equitable. Journal of Intelligence.

[B27-jintelligence-12-00129] Hommel Björn E., Ruppel Regina, Zacher Hannes (2022). Assessment of cognitive flexibility in personnel selection: Validity and acceptance of a gamified version of the Wisconsin Card Sorting Test. International Journal of Selection and Assessment.

[B28-jintelligence-12-00129] Hox Joop J. (2010). Multilevel Analysis: Techniques and Applications.

[B29-jintelligence-12-00129] Jones Marshall B., Dunlap William P., Bilodeau Ina McD. (1986). Comparison of video game and conventional test performance. Simulation & Games.

[B30-jintelligence-12-00129] Kantrowitz Tracy M., Gutierrez Sara L., Scott John C., Bartram Dave, Reynolds Douglas H. (2018). The changing landscape of technology-enhanced test administration. Next Generation Technology-Enhanced Assessment.

[B31-jintelligence-12-00129] Koch Marco, Becker Nicolas, Spinath Frank M., Greiff Samuel (2021). Assessing intelligence without intelligence tests. Future perspectives. Intelligence.

[B32-jintelligence-12-00129] Krebs Christine, Falkner Michael, Niklaus Joel, Persello Luca, Klöppel Stefan, Nef Tobias, Urwyler Prabitha (2021). Application of Eye Tracking in Puzzle Games for Adjunct Cognitive Markers: Pilot Observational Study in Older Adults. JMIR Serious Games.

[B33-jintelligence-12-00129] Kuncel Nathan R., Hezlett Sarah A., Ones Deniz S. (2004). Academic performance, career potential, creativity, and job performance: Can one construct predict them all?. Journal of Personality and Social Psychology.

[B34-jintelligence-12-00129] Landers Richard N., Sanchez Diana R. (2022). Game-based, gamified, and gamefully designed assessments for employee selection: Definitions, distinctions, design, and validation. International Journal of Selection and Assessment.

[B35-jintelligence-12-00129] Landers Richard N., Armstrong Michael B., Collmus Andrew B., Mujcic Salih, Blaik Jason T. (2021). Theory-driven game-based assessment of general cognitive ability: Design theory, measurement, prediction of performance, and test fairness. Journal of Applied Psychology.

[B36-jintelligence-12-00129] Landers Richard N., Bauer Kristina N., Callan Rachel C. (2017). Gamification of task performance with leaderboards: A goal setting experiment. Computers in Human Behavior.

[B37-jintelligence-12-00129] Lang Jonas W. B., Kell Harrison J. (2020). General mental ability and specific abilities: Their relative importance for extrinsic career success. Journal of Applied Psychology.

[B38-jintelligence-12-00129] Lievens Filip, De Soete Britt, Schmitt N. (2012). Simulations. Handbook of Assessment and Selection.

[B39-jintelligence-12-00129] Lievens Filip, Reeve Charlie L. (2015). Where I–O Psychology Should Really (Re)start Its Investigation of Intelligence Constructs and Their Measurement. Industrial and Organizational Psychology.

[B40-jintelligence-12-00129] Lievens Filip, Sackett Paul R. (2017). The effects of predictor method factors on selection outcomes: A modular approach to personnel selection procedures. Journal of Applied Psychology.

[B41-jintelligence-12-00129] Lumsden Jim, Edwards Elizabeth A., Lawrence Natalia S., Coyle David, Munafò Marcus R. (2016). Gamification of Cognitive Assessment and Cognitive Training: A Systematic Review of Applications and Efficacy. JMIR Serious Games.

[B42-jintelligence-12-00129] Malanchini Margherita, Rimfeld Kaili, Gidziela Agnieszka, Cheesman Rosa, Allegrini Andrea G., Shakeshaft Nicholas, Schofield Kerry, Packer Amy, Ogden Rachel, McMillan Andrew (2021). Pathfinder: A gamified measure to integrate general cognitive ability into the biological, medical, and behavioural sciences. Molecular Psychiatry.

[B43-jintelligence-12-00129] McGrew Kevin S. (2009). CHC theory and the human cognitive abilities project: Standing on the shoulders of the giants of psychometric intelligence research. Intelligence.

[B44-jintelligence-12-00129] Mead Alan D., Drasgow Fritz (1993). Equivalence of computerized and paper-and-pencil cognitive ability tests: A meta-analysis. Psychological Bulletin.

[B45-jintelligence-12-00129] Melchers Klaus G., Basch Johannes M. (2022). Fair play? Sex-, age-, and job-related correlates of performance in a computer-based simulation game. International Journal of Selection and Assessment.

[B46-jintelligence-12-00129] Nye Christopher D., Ma Jingjing, Wee Serena (2022). Cognitive Ability and Job Performance: Meta-analytic Evidence for the Validity of Narrow Cognitive Abilities. Journal of Business and Psychology.

[B47-jintelligence-12-00129] Ohlms Marie L., Melchers Klaus G., Kanning Uwe P. (2024). Can we playfully measure cognitive ability? Construct-related validity and applicant reactions. International Journal of Selection and Assessment.

[B48-jintelligence-12-00129] Ones Deniz S., Dilchert Stephen, Viswesvaran Chockalingam, Schmitt N. (2012). Cognitive abilities. The Oxford Handbook of Personnel Assessment and Selection.

[B49-jintelligence-12-00129] Quiroga Maria A., Escorial Sergio, Román Francisco J., Morillo Daniel, Jarabo Andrea, Privado Jesús, Hernández Miguel, Gallego Borja, Colom Roberto (2015). Can we reliably measure the general factor of intelligence (g) through commercial video games? Yes, we can!. Intelligence.

[B50-jintelligence-12-00129] Quiroga Maria A., Diaz Alice, Román Francisco J., Privado Jesús, Colom Roberto (2019). Intelligence and video games: Beyond “brain-games”. Intelligence.

[B51-jintelligence-12-00129] Quiroga Maria A., Román Francisco J., Fuente Javier De La, Privado Jesús, Colom Roberto (2016). The measurement of intelligence in the XXI century using video games. The Spanish Journal of Psychology.

[B52-jintelligence-12-00129] Quiroga Maria. A., Herranz María, Gómez-Abad Marta, Kebir Muna, Ruiz Javier, Colom Roberto (2009). Video-games: Do they require general intelligence?. Computers & Education.

[B53-jintelligence-12-00129] Quiroga Maria A, Román Francisco J., Catalán Ana, Rodríguez Herman, Ruiz Javier, Herranz María, Gómez-Abad Marta, Colom Roberto (2011). Videogame Performance (Not Always) Requires Intelligence. International Journal of Online Pedagogy and Course Design.

[B54-jintelligence-12-00129] Rabbitt Patrick, Banerji Nicole, Szymanski Alex (1989). Space Fortress as an IQ test? Predictions of learning and of practised performance in a complex interactive video-game. Acta Psychologica.

[B55-jintelligence-12-00129] Ramos-Villagrasa Pedro J., Fernández-del-Río Elena, Castro Ángel (2022). Game-related assessments for personnel selection: A systematic review. Frontiers in Psychology.

[B56-jintelligence-12-00129] Roman Franciso J., Gutierrez Pablo, Ramos-Cejudo Juan, Gonzalez-Calero Pedro A., Gomez-Martin Pedro P., Larroy Cristina, Martin-Brufau Ramon, Lopez-Cavada Carlos, Quiroga Maria A. (2024). Checking Different Video Game Mechanics to Assess Cognitive Abilities in Groups with and without Emotional Problems. Journal of Intelligence.

[B57-jintelligence-12-00129] Sackett Paul R., Zhang Charlene, Berry Christopher M., Lievens Filip (2022). Revisiting meta-analytic estimates of validity in personnel selection: Addressing systematic overcorrection for restriction of range. Journal of Applied Psychology.

[B58-jintelligence-12-00129] Sackett Paul R., Lievens Filip, Iddekinge Chad H. Van, Kuncel Nathan R. (2017). Individual differences and their measurement: A review of 100 years of research. Journal of Applied Psychology.

[B59-jintelligence-12-00129] Sala Giovanni K., Tatlidil Semir, Gobet Fernand (2018). Video game training does not enhance cognitive ability: A comprehensive meta-analytic investigation. Psychological Bulletin.

[B60-jintelligence-12-00129] Sanchez Diana R., Weiner Erik, Zelderen Anand Van (2022). Virtual reality assessments (VRAs): Exploring the reliability and validity of evaluations in VR. International Journal of Selection and Assessment.

[B61-jintelligence-12-00129] Schmidt Frank L., Hunter John E. (2015). Methods of Meta-Analysis.

[B62-jintelligence-12-00129] Schneider W. Joel, McGrew Kevin, Flanagan Dwan P., McDonough Erin M. (2012). The Cattell-Horn-Carroll model of intelligence. Contemporary Intellectual Assessment: Theories, Tests, and Issues.

[B63-jintelligence-12-00129] Stanek Kevin C., Ones Deniz S., Ones Deniz S., Anderson Neal, Viswesvaran Chockalingam, Sinangil Handan K. (2018). Taxonomies and compendia of cognitive ability and personality constructs and measures relevant to industrial, work and organizational psychology. The SAGE Handbook of Industrial, Work & Organizational Psychology: Personnel Psychology and Employee Performance.

[B64-jintelligence-12-00129] Sternberg Robert J., Sternberg R. J. (2020). Approaches to understand human intelligence. Intelligence.

[B65-jintelligence-12-00129] Tippins Nancy T., Beaty James, Drasgow Fritz D., Gibson Wade M., Pearlman Kenneth, Segall Daniel, Shepherd William J. (2006). Unproctored, internet testing in employment settings. Personnel Psychology.

[B66-jintelligence-12-00129] Tippins Nancy T., Oswald Frederick L., McPhail S. Morton (2021). Scientific, Legal, and Ethical Concerns About AI-Based Personnel Selection Tools: A Call to Action. Personnel Assessment and Decisions.

[B67-jintelligence-12-00129] Traylor Zach, Hagen Ellen, Williams Ashleigh, Arthur Winfred (2021). The testing environment as an explanation for unproctored internet-based testing device-type effects. International Journal of Selection and Assessment.

[B68-jintelligence-12-00129] van de Schoot Rens, Bruin Jonathan de, Schram Raoul, Zahedi Parisa, Boer Jan de, Weijdema Felix, Kramer Bianca, Huijts Martijn, Hoogerwerf Maarten, Ferdinands Gerbrich (2021). An open source machine learning framework for efficient and transparent systematic reviews. Nature Machine Intelligence.

[B69-jintelligence-12-00129] van Lill Xander, McColl Laird, Neale Matthew (2023). Cross-national applicability of a game-based cognitive assessment. International Journal of Selection and Assessment.

[B70-jintelligence-12-00129] Viechtbauer Wolfgang (2010). Conducting meta-analyses in R with the metafor package. Journal of Statistical Software.

[B71-jintelligence-12-00129] Wahlstrom Dustin, Raiford Susan E., Breaux Kristina C., Zhu Jianjun, Weiss Lawrence G., Flanagan Dwan P., McDonough Erin M. (2012). The Wechsler Preschool and primary scacle of intelligence—Forth edition. Contemporary Intellectual Assessment: Theories, Tests, and Issues.

[B72-jintelligence-12-00129] Weinder Nathan, Short Elisabeth, Landers Richard N. (2018). Playing with a purpose: The role of games and gamification in modern assessment practices. The Cambridge Handbook of Technology and Employee Behavior.

[B73-jintelligence-12-00129] Weiner Erik J., Sanchez Diana R. (2020). Cognitive ability in virtual reality: Validity evidence for VR game-based assessments. International Journal of Selection and Assessment.

[B74-jintelligence-12-00129] Wu Felix Y., Mulfinger Evan, III Leo Alexander, Sinclair Andrea L., McCloy Rodney A., Oswald Frederick L. (2022). Individual differences at play: An investigation into measuring Big Five personality facets with game-based assessments. International Journal of Selection and Assessment.

